# Characterization of a 3S PRAME VLD-Specific T Cell Receptor and Its Use in Investigational Medicinal Products for TCR-T Therapy of Patients with Myeloid Malignancies

**DOI:** 10.3390/cancers17020242

**Published:** 2025-01-13

**Authors:** Maja Bürdek, Petra U. Prinz, Kathrin Mutze, Stefanie Tippmer, Christiane Geiger, Giulia Longinotti, Dolores J. Schendel

**Affiliations:** 1 Medigene Immunotherapies GmbH, 82152 Planegg-Martinsried, Germany; p.prinz@medigene.com (P.U.P.); k.mutze@medigene.com (K.M.); s.tippmer@medigene.com (S.T.); c.geiger@medigene.com (C.G.); g.longinotti@medigene.com (G.L.); 2 Medigene AG, 82152 Planegg-Martinsried, Germany

**Keywords:** TCR-T therapy, PReferentially expressed Antigen in MElanoma, investigational medicinal products, preclinical studies, acute myeloid leukemia, myelodysplastic syndrome, multiple myeloma, blood cancers

## Abstract

Background/Objectives: MDG1011 is an autologous TCR-T therapy developed as a treatment option for patients with myeloid malignancies, including acute myeloid leukemia (AML), myelodysplastic syndrome (MDS), and multiple myeloma (MM). It is specific for the target antigen PReferentially expressed Antigen in MElanoma (PRAME). The recombinant TCR used in MDG1011 recognizes PRAME_100–108_ VLD-peptide presented by HLA-A*02:01-encoded surface molecules. Methods: Two preclinical batches of MDG1011, produced from enriched CD8+ T cells of healthy donors, underwent rigorous evaluation of on-target and off-target recognition of tumor cells and test cells representing healthy tissues. MDG1011 investigational medicinal products (IMPs) were produced for 13 patients. VLD-TCR surface expression was assessed using dual-marker flow cytometry using TCR V-beta-specific antibody and VLD/HLA-A2-specific multimer. Functionality was assessed by interferon-gamma (IFN-γ) secretion and cell-mediated cytotoxicity of target cells. Results: Preclinical MDG1011 batches displayed strong VLD-TCR expression, cytokine secretion, and cytotoxicity after antigen-specific activation, while showing no signals of on-target/off-tumor or off-target recognition. All IMPs had good VLD-TCR expression as well as functionality after activation by multiple target cells. Conclusions: Preclinical studies demonstrated that MDG1011 displayed key 3S attributes of high specificity, sensitivity, and safety required for regulatory approval of a first-in-human (FIH) clinical study of patients with myeloid malignancies (CD-TCR-001: ClinicalTrials.gov Identifier: NCT03503968). MDG1011 IMP manufacturing was successful at 92%, even including heavily pretreated elderly patients with very advanced disease. The IMPs applied in nine patients all displayed antigen-specific functionality. Elsewhere, clinical study results for MDG1011 showed no dose-limiting toxicity and signs of biological and/or clinical activity in several patients.

## Simple Summary

TCR-T therapies have the potential to capture the power of adoptive cell therapy, as has been successfully developed for CAR-T therapies for several B cell malignancies and multiple myeloma. CAR-T therapies for myeloid malignancies are hindered by lack of safe target antigens and healthy cell toxicity. Therefore, TCR-T therapies recognizing other targets are explored for these blood cancers. The antigen PReferentially expressed Antigen in MElanoma, PRAME, was shown to be a T cell target for acute myeloid leukemia after stem cell transplantation, without overt toxicity. Therefore, PRAME-specific TCR-T therapies may serve well for treatment of AML and myelodysplastic syndrome. PRAME-specific TCR-T therapy may also be suited for relapsed multiple myeloma after CAR-T therapy, arising from outgrowth of malignant cells due to loss of CAR-T antigens. On this basis, MDG1011, a PRAME-specific TCR-T therapy, was developed to fill an unmet medical need for new treatment options for these myeloid malignancies.

## 1. Introduction

Chimeric antigen receptor (CAR)-T cells used in adoptive cell therapy (ACT) provide patients with millions to billions of T cells that recognize and kill tumor cells through the expression of antibody-derived receptors that directly bind surface proteins on cancer cells. The power of CAR-T therapies was demonstrated in multiple studies showing high efficacy with long-term clinical benefit in many patients with blood cancers, even potential cure after a single treatment [1]. Currently, six CAR-T therapies are commercially available in different countries to treat diverse B lymphoid malignancies and multiple myeloma (MM), based on CAR-T cells specific for the target antigens, CD19 [2,3,4,5], and B cell membrane antigen (BCMA) [6,7]. Newer approaches apply CAR-T therapies in solid cancers [8] and, most recently, autoimmune diseases [9].

ACT is also explored using T cells that are engineered to express T cell receptors (TCRs) that recognize antigenic peptides presented by human leukocyte antigen (HLA) molecules (i.e., known as HLA-restriction) for both liquid and solid cancers [10,11]. The US Food and Drug Administration recently approved *afamitresgene autoleucel* (TECELRA^®^), directed against the cancer-testis antigen (CTA) MAGE-A4, for treatment of synovial sarcoma and myxoid/round cell liposarcoma [12,13].

Many ongoing clinical studies of TCR-T therapy of blood cancers are based on specific antigens identified through T cell responses associated with successful hematopoietic stem cell transplantation [14]. One of these is the CTA PReferentially Expressed Antigen in MElanoma (PRAME). PRAME has several features that support its use as a target antigen for several types of blood cancer [15]. Most importantly, PRAME is frequently expressed in leukemic cells of myeloid and lymphoid origin but displays no or very limited expression in healthy tissues. This gives PRAME a highly differentiated target safety profile. A central function of PRAME as a regulator of hematopoietic differentiation and apoptosis [16] may provide leukemic cells with survival advantages and guard against immune selection of antigen-loss variants that can hinder clinical efficacy. Previous studies showed PRAME to be a suitable target for HLA-restricted T cell responses in vitro and in vivo [17,18,19]. Furthermore, PRAME-specific T cells were associated with long-term survival of patients with acute myeloid leukemia (AML) after allogeneic stem cell transplantation [20].

On this basis, PRAME-specific TCRs were isolated from antigen-specific T cells of HLA-A*02:01-positive healthy donors, according to published details [21]. They were screened for the key 3S characteristics of high specificity, sensitivity, and safety, which are essential attributes for clinical development of TCR-engineered therapies [22]. TCR T4.8-1-29, recognizing the PRAME_100–108_ peptide epitope (hereafter VLD), was selected as the lead 3S TCR for development of MDG1011, an autologous TCR-T therapy designed for patients with blood cancers known to express PRAME, including relapsed or refractory (R/R) AML, myelodysplastic syndrome (MDS), and MM. Following development and regulatory approval of a GMP-compliant TCR-T production process based on enriched CD8+ T cells, investigational medicinal products (IMPs) were manufactured for individual patients entered in a FIH clinical study of MDG1011 TCR-T therapy.

Here, we present details of the extensive preclinical evaluation of MDG1011 with respect to its 3S attributes, using cells prepared from healthy donors. Thereafter, we show characterization studies of MDG1011 IMPs manufactured for individual patients included in the first part of the Phase I/II multicenter, non-randomized, open-label clinical trial [CD-TCR-001: Eudra CT 2017-000440-18] evaluating MDG1011 TCR-T therapy. General outcomes of the Phase I part of this clinical trial have been presented elsewhere, demonstrating the lack of dose-limiting toxicity, and indicating biological and/or clinical activity in several patients. Clinical observations were supported by the continued presence of MDG1011 cells in peripheral blood (PB) and by decreases in PRAME mRNA levels in either PB or bone marrow (BM) [23].

## 2. Materials and Methods

### 2.1. Isolation of VLD-TCR T4.8-1-29 and Generation of VLD-TCR-T Cells (MDG1011)

To obtain TCRs with defined antigen specificity for potential clinical development, the few naturally occurring T cells within PB of healthy human donors with specificity for epitopes derived from the target antigen PRAME were activated (primed) in an autologous priming approach using dendritic cells (DC) transfected with PRAME-encoding in vitro transcribed (*ivt*)RNA [24]. T cells activated by PRAME protein-derived epitopes, in complexes with HLA-A*02:01-encoded surface molecules, were expanded by adding interleukin (IL)-2 and IL-7 to the culture medium. T cells expressing TCRs specific for the PRAME VLD-peptide, VLDGLDVLL, were isolated using a multimer-based flow cytometry staining procedure and separated by single cell cloning using fluorescence-activated cell sorting (FACS) technology.

TCR-α and TCR-β chains of PRAME VLD-reactive T cell clones were identified by next-generation sequencing (NGS), as described elsewhere [25]. TCR chain constant regions were minimally murinized to increase transgenic TCR heterodimer stability [24]. Codon-optimized TCR chains, linked by a P2A peptide and carrying a cysteine disulfide bond [26], were cloned into the pES.12-6 self-inactivating (SIN) gamma-retrovirus vector.

Leukapheresis material of two healthy donors (Donor_A and Donor_B) was provided by the Stefan-Morsch-Stiftung (Birkenfeld/Nahe, Germany) and used for the generation of T4.8-1-29-expressing TCR-T cells (VLD-TCR-T cells). CD8+ cells were enriched using positive immuno-magnetic separation and the CliniMACS^®^ system (Miltenyi Biotec, Bergisch-Gladbach, Germany) at the contract development and manufacturing organization (CDMO) BioNTech IMFS GmbH (Idar-Oberstein, Germany). SIN-γ retroviral supernatant encoding T4.8-1-29 (harvested from a producer cell line under research and development conditions, but according to procedures established for the GMP process at the CDMO (BioNTech) was used for transduction of CD8+ T cells. Un-transduced (UT) T cells were cultured in parallel as controls. The cells were expanded until day 10, harvested, cryopreserved, and stored at −150 °C. Allo-A2-reactive TCR-T cells capable of recognizing any HLA-A*02:01-positive target cell were used as controls and generated from CD8+ T cells of one healthy donor (Donor_A) using R&D retroviral supernatant encoding T5.8-3-9 (produced at Medigene, Planegg-Martinsried, Germany). After expansion, CD8/T5.8-3-9-TCR double-positive cells were sorted using a FACSAria™ cell sorter (BD Biosciences, Franklin Lakes, NJ, USA), re-stimulated, and cryopreserved at day 13 after restimulation.

Prior to use in nonclinical assays, the cells were thawed, and the percentage of TCR-transgenic T cells was determined by flow cytometry using fluorochrome-labeled CD8- and TRBV9-specific antibodies (anti-CD8: clone RPA-T8, BD Biosciences; anti-TRBV9: clone BL37.2, Beckman Coulter, Brea, CA, USA). Percentages of TRBV9-positive CD8+ T cells, above endogenous TRBV9 levels detected in UT cells, were considered to represent the percentage of CD8+ T cells carrying the transgenic TCR. Transgenic expression of the allo-A2-reactive TCR T5.8-3-9 in CD8+ T cells was detected with an anti-murine Cβ antibody (Clone H57-597, BD Biosciences), since the TCR was reconstructed using a murine C region.

### 2.2. Cell Culture of Target Cell Lines

T2 cells (German Collection of Microorganisms and Cell Cultures GmbH—DSMZ, Braunschweig, Germany), acquired lymphoblastoid cell lines (LCLs) (International Histocompatibility Working Group, Fred Hutchinson Cancer Research Center, Seattle, WA, USA), or generated in-house by transduction of Donor B cells with Epstein–Barr virus (EBV) strain B95.8 virus-containing supernatant (kind gift of A. Moosmann, Helmholtz Zentrum München, Germany), Mel624.38 (kind gift of M. Panelli, National Institutes of Health, Bethesda, MD, USA), MelA375, and K562 (American Type Culture Collection—ATCC, Manassas, VI, USA), were maintained in RPMI 1640 with 10% FCS and supplemented as described [21]. All cell lines were confirmed to be mycoplasma-free by PCR (Venor^®^GeM Classic, Minerva Biolabs, Berlin, Germany) prior to use in any experiment.

Primary HLA-A*02:01-positive human renal cortical epithelial cells (HRCEpC, PromoCell, Heidelberg, Germany), normal human lung fibroblasts (NHLF, Lonza, Basel, Switzerland), and induced pluripotent stem cell-derived iCell Cardiomyocytes (Cardio), iCell endothelial cells (EC), iCell Hepatocytes (Hep), iCell Astrocytes (Astro), and iCell Neurons (Neuro) (FujiFilm CDI, Madison, WI, USA) were cultured according to the manufacturers’ instructions. Mature dendritic cells (mDC) were generated from HLA-A*02:01-positive healthy donor monocytes isolated from peripheral blood mononuclear cells (PBMC) [27]. Primary normal human bronchial epithelial cells (NHBE, Lonza), endogenously negative for HLA-A*02:01, were electroporated using *ivt*RNA encoding HLA-A*02:01.

### 2.3. Quantitative Real-Time PCR (qPCR)

Expression levels of PRAME mRNA in different cell lines were determined by qPCR. For this, cDNA was generated from the cell samples using the Transcriptor First Strand cDNA kit (Roche, Basel, Switzerland) and subsequently analyzed with the Real-Time PCR System Light Cycler 480 (Roche). The house-keeping gene GUSB served as the control and was used for normalization.

### 2.4. Cytokine Release Assay

IFN-γ or IL-2 secretion by CD8+ T cells after co-culture with different target cells was assessed in supernatant media after 24 h. Cytokine concentrations were determined using ELISA kits (Thermo Fisher Scientific, Waltham, MA USA, 88-7316-77; Becton Dickinson, 555142; or Becton Dickinson, 555190, Franklin Lakes, NJ, USA). The OD measurement was performed using a Multiskan Microplate-Photometer (Thermo Fisher Scientific). Background-corrected OD values were used for extrapolation using a third-degree polynomial.

### 2.5. Live-Cell Imaging Cytotoxicity Assay

Cell killing was assessed using an IncuCyte ZOOM^®^ device (Sartorius, Göttingen, Germany) following manufacturer’s recommendations for real-time quantitative live-cell imaging. Cells were seeded into 96-well flat-bottom plates at least 24 h prior to the addition of CD8+ TCR-T cells, and target cell lysis was monitored by plate scanning every 2 or 4 h. Tumor cell lines were transduced to express the nuclear-restricted red fluorescent protein, mKate2, for easier visualization of tumor cell killing through detection of decreases in red fluorescence over time. For IMP characterization, the percentage of target cell killing was calculated in relation to UT controls. Healthy cells were tested without prior manipulation to maintain physiological gene expression. Phase contrast images of healthy cells were evaluated by a panel of experienced internal scientists for changes in target cell layer confluency and cell morphology, indicating the killing of target cells.

### 2.6. Flow Cytometry

Immune cell subtype composition of starting apheresis material, intermediate CD8+ T cells, and MDG1011 IMPs was investigated by flow cytometry with fluorochrome-labeled antibodies (Multitest CD3/16 and 56/45/19, Multitest CD3/8/45/4, BD Biosciences, San Jose, CA, USA; anti-CD14: clone 61D3, Thermo Fisher Scientific; anti-CD34: clone 8G12, BD Biosciences). Cell staining patterns were acquired using a FACSVerse flow cytometer (BD Biosciences).

For analysis of T-memory markers, cells were stained with fluorochrome-labeled antibodies, including anti-CD45 (clone HI30), anti-CD3 (clone UCHT1), anti-CD8 (clone RPA-T4), anti-CD27 (clone L128), anti-CD95 (clone DX2), and anti-CCR7 (clone 150503), all purchased from BD Biosciences, and anti-CD45-RA (clone HI100) purchased from eBioscience (Frankfurt, Germany). Cell staining patterns were acquired using the LSR Fortessa flow cytometer (BD Biosciences), and data were analyzed using FlowJo software version 10 (BD Biosciences). For analysis of memory CD8+ T cells, CD45RA+/CCR7+/CD27+/CD95− T cells were defined as naïve T cells (Tn), CD45RA+/CCR7+/CD27+/CD95+ T cells as stem cell memory T cells (Tscm), CD45RA−/CCR7+/CD27+ T cells as central memory T cells (Tcm), CD45RA−/CCR7−/CD27− T cells as effector memory T cells (Tem), and CD45RA+/CCR7−/CD27− T cells as effector memory T cells RA-positive (Temra)

The expression of the transgenic TCR in IMP samples was analyzed by flow cytometry using fluorochrome-labeled antibodies, including anti-CD45 (clone HI30), anti-CD3 (clone UCHT1), anti-CD8 (clone RPA-T4), all purchased from BD Biosciences, and anti-TRBV9-specific antibodies (clone BL37.2) from Beckman Coulter, as well as the HLA-A*02:01/PRAME-VLD dextramer (Immudex, Copenhagen, Denmark). Cell acquisition was performed on an LSR Fortessa, and the resulting data were analyzed using FlowJo software (BD Biosciences).

## 3. Results

### 3.1. Preclinical Studies of MDG1011 Effector Cells of Healthy Donors

#### 3.1.1. Surface Expression of Recombinant VLD-TCR T4.8-1-29 in CD8-Enriched T Cells

**Figure 1:**
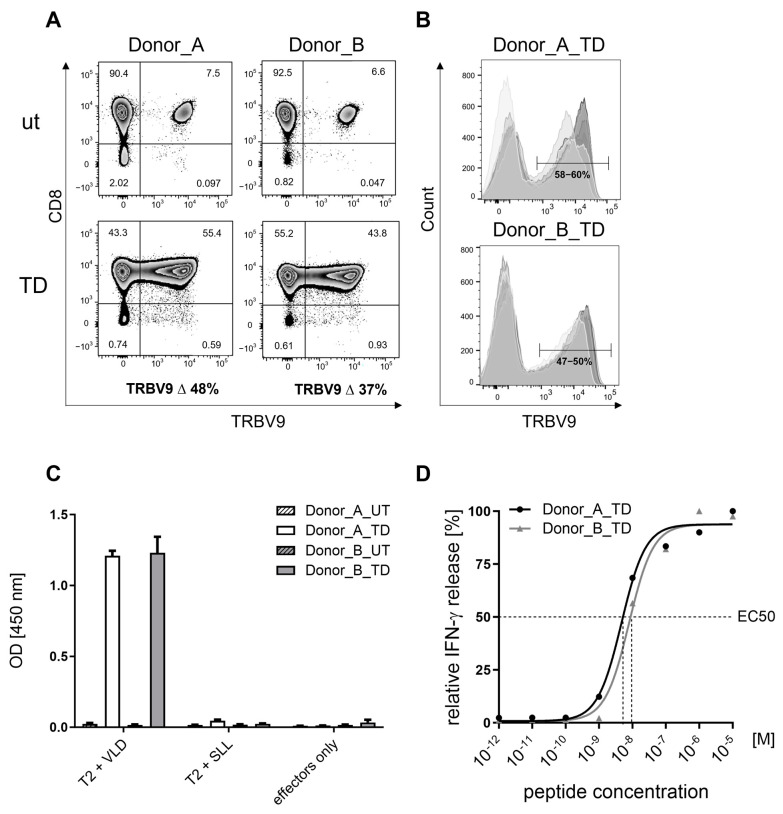
Cell surface VLD-TCR expression and PRAME VLD specificity of MDG1011. (**A**) Surface expression of VLD-TCR on CD8+ T cells was determined at day 10 after stimulation. CD8+ T cells of Donor_A and Donor_B were transduced with retrovirus encoding the VLD-TCR and expression in transduced (TD) cells was determined using flow cytometry using an anti-TRBV9 antibody (clone BL37.2, Beckman Coulter) and calculated by subtraction of the measured percentage of TRBV9 expression in UT CD8+ T cells, representing the endogenous TRBV9 expression in the respective donor. (**B**) Histograms show an overlay of eight independent analyses of VLD-TCR expression on T cells of both donors using different shades of gray. T cells were stained with CD8-specific and TRVB9-specific antibodies and were analyzed using flow cytometry. The cell populations in the histograms are pre-gated on lymphocytes (FSC-A/SSC-A), single cells (FSC-A/FSC-H), and CD8+ cells. (**C**) Specific recognition of PRAME-VLD-loaded T2 cells. UT and VLD-TCR-expressing T cells of both donors were co-cultured with T2 cells, loaded either with PRAME-VLD (VLD) or irrelevant PRAME-SLL (SLL) (10^−5^ M each) as control. Supernatants were harvested after 24 h and IFN-γ content analyzed using ELISA. T cells without T2 target cells (effectors only) were used as negative control. Shown are mean values of triplicates with standard deviations. (**D**) Functional avidity of VLD-TCR-expressing T cells. VLD-TCR-transduced T cells derived from Donor_A (black line) and Donor_B (gray line) were co-cultured with T2 cells loaded with titrated amounts of PRAME-VLD (VLD), ranging from 10^−5^ M to 10^−13^ M. Supernatants were harvested after 24 h and IFN-γ content analyzed using ELISA. Dashed lines indicate the peptide concentrations required to induce half maximal IFN-γ secretion (EC50).

Lymphocytes enriched for CD8+ T cells from leukapheresis starting materials of healthy donors A and B were transduced with the PRAME VLD-TCR to assess specificity, sensitivity, and safety. UT batches of both donors served as VLD-TCR-negative control effector cells. Cryopreserved UT and MDG1011 cells were used for subsequent preclinical studies without pre-activation. After thawing, they were assessed for TCR surface expression in CD8+ T cells by flow cytometry using a TRBV9-specific antibody, which binds VLD-TCRs as well as endogenous TRBV9-TCRs expressed by a variable fraction of T cells. Around 6–8% of UT and 47–60% of VLD-TCR-transduced (TD) effector cells bound TRBV9-specific antibody (Figure 1A). After subtraction of respective UT values, it was determined that between 40 and 47% of the MDG1011 batches of donors A and B expressed VLD-TCRs, respectively. An overlay of eight independent analyses showed consistent and stable patterns of TRBV9-staining in MDG1011 batches of both donors after thawing, representative of the effector cells used for subsequent functional studies (Figure 1B). Control allo-A2 TCR-T cells, prepared from donor A to stably express the HLA-A2-allo-reactive TCR T5.8-3-9, were analyzed using an anti-murine Cβ antibody, showing transgenic TCR expression on 71% of CD8+ T cells (internal unpublished data).

#### 3.1.2. Specificity of MDG1011

PRAME specificity of MDG1011 was assessed by IFN-g secretion after stimulation with PRAME peptide-pulsed antigen-presenting cells (APCs). UT and MDG1011 batches were co-cultured with HLA-A2-positive T2 cells used as APCs after pulsing with exogenous synthetic peptides. PRAME_100–108_ (VLDGLDVLL) was the specific test peptide, and PRAME_425–433_ peptide (SLLQHLIGL) was a negative control peptide, known to bind well to HLA-A2 molecules and stimulate PRAME-specific SLL-TCR-expressing T cells [21]. Unloaded T2 cells served as negative control APCs. Co-culture supernatants harvested at 24 h were analyzed for IFN-γ using an ELISA. Both MDG1011 batches only secreted IFN-γ in co-cultures with T2 cells pulsed with VLD, demonstrating their clear VLD specificity (Figure 1C). UT batches did not respond to these APCs, showing that VLD-TCR expression by MDG1011 effector cells was required for specific recognition. Elsewhere, HLA-A2-negative PRAME peptide-pulsed APCs failed to induce MDG1011 IFN-γ secretion, confirming the HLA-A2-restricted presentation of VLD.

#### 3.1.3. Sensitivity of MDG1011

MDG1011 peptide sensitivity was assessed in a functional avidity assay measuring IFN-γ secretion after stimulation by T2 cells pulsed with serial dilutions of PRAME_100–108_ peptide, ranging from 10^−5^ to 10^−13^ M of peptide. The two MDG1011 batches displayed comparable sensitivity, represented by concentrations of VLD that elicited half-maximal capacity for secretion of IFN-γ (EC50 values), determined using an ELISA of co-culture supernatants at 24 h (Figure 1D). EC50 values were near 1 × 10^−8^ M for the two MDG1011 batches.

#### 3.1.4. Cytotoxic Activity of MDG1011

**Figure 2:**
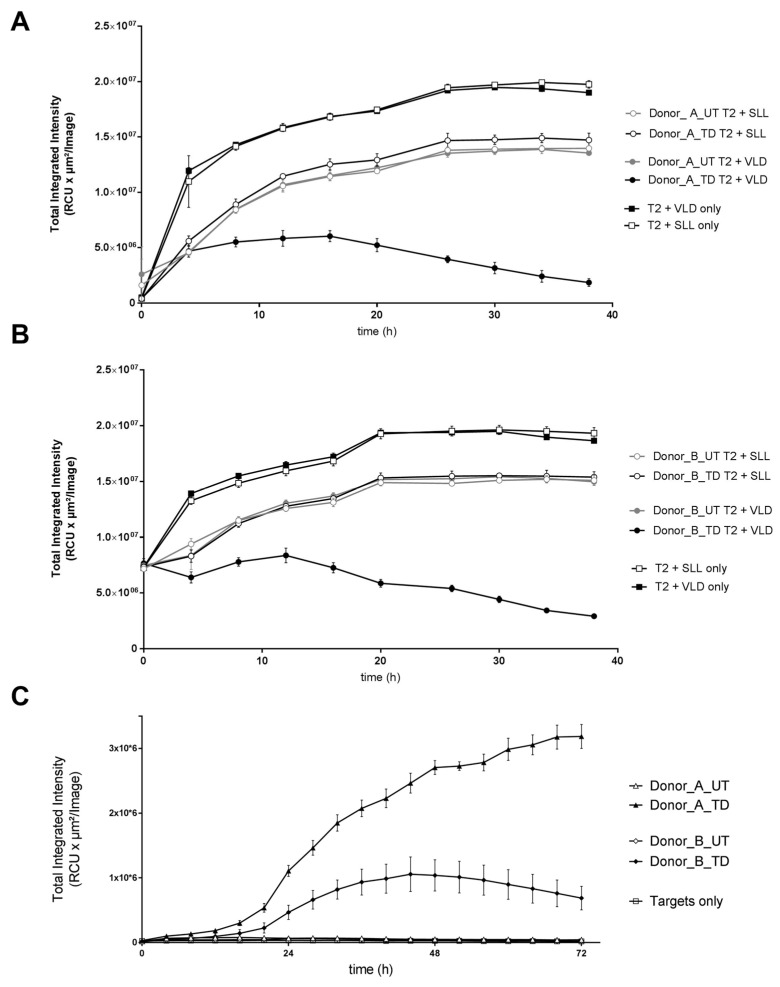
MDG1011-mediated killing of PRAME VLD-positive target cells. Red fluorescent T2 cells (mKate2-transduced) were loaded either with target VLD or irrelevant control SLL (10^−5^ M each). UT or VLD-TCR-transduced (TD T cells derived from Donor_A (**A**) and Donor_B (**B**) were added, and images were taken every 4 h for up to 38 h. Shown is the reduction in total integrated intensity (*y*-axis) of the fluorescence-labeled target cells over time (*x*-axis). Lower levels of fluorescence intensity in combinations of UT cells with PRAME VLD-pulsed T2 cells and MDG1011 with PRAME SLL-pulsed T2 cells, compared to T2 cells alone, may reflect signal quenching by T cells. (**C**) The MElanoma cell line Mel624.38 (HLA-A*02-positive/PRAME-positive), seeded in 96-well flat-bottom plates, was co-cultured with UT CD8+ T cells derived from Donor_A (white triangle) or Donor_B (white diamond) or VLD-TCR-transduced T cells derived from Donor_A (black triangle) or Donor_B (black diamond) for 72 h. A red fluorescent annexin V dye was added to the culture medium to visualize tumor cell apoptosis. Mel624.38 cells without T cells (targets only) and UT CD8+ T cells served as negative controls. Shown are the total integrated fluorescence intensities in RCU × µm^2^/image over time.

MDG1011 capacity to mediate target cell killing was tested in cytotoxicity assays using two different target cells. In the first case, T2 cells were fluorescently labeled for live cell tracking using the IncuCyte ZOOM^®^ imaging system. Labeled T2 target cells were pulsed exogenously with either VLD or SLLs, washed and co-cultured for 38 h with UT and MDG1011 T cells and images were taken every 4 h for the duration of the experiment. Results are shown in Figure 2A,B for donors A and B, respectively, plotting time against reduction in total integrated intensity of fluorescence of labeled T2 target cells. Strong reductions were only seen with MDG1011 T cells combined with VLD-pulsed targets, reflecting concomitant target cell loss over time. Importantly, intensity changes were not seen with MDG1011 combined with SLL-pulsed APCs. Furthermore, no reductions were observed for UT cells with VLD-pulsed APCs, confirming dependency of target killing on VLD-TCR expression.

MDG1011-mediated cytotoxicity of tumor cells was determined using Mel624.38 target cells that co-express natural levels of HLA-A2 and endogenous intracellular PRAME proteins. Both MDG1011 batches recognized Mel624.38 cells, which led to an uptake of red fluorescent annexin V dye that demarks apoptotic cells, which increased over 72 h of live cell imaging. No apoptosis was induced by UT cells, confirming dependency for VLD-TCR expression for MDG1011 tumor cell recognition (Figure 2C).

#### 3.1.5. Safety Profile of MDG1011

Safety was of paramount importance in judging the suitability of MDG1011 for clinical development. Safety assessments were complex and entailed studies of cytokine secretion and cell-mediated cytotoxicity using multiple test cell panels. Due to the extensive scope of these studies, the results of four critical assessments are summarized in Table 1. Experiments addressed on-target/off-tumor recognition of HLA-A2/PRAME double-positive target cells and off-target toxicity against HLA-A2-positive test cells that did not express PRAME, using a cell panel representing vital healthy tissues. Other experiments tested MDG1011 recognition of synthetic peptides presented by T2 cells, which were partially mismatched to the PRAME_100–108_ sequence and identified in the human exome or proteome by in silico technologies [28]. MDG1011 cross-recognition of HLA molecules other than those encoded by the HLA-A*02:01 allele was assessed with a test panel of lymphoblastoid cell lines (LCL) representing diverse HLA allotypes. Examples of these assessments have been published previously [21].

Exemplary data of one safety assessment, included in Table 1, are shown for UT and MDG1011 cells co-cultured with an HLA-A*02:01-positive test panel comprising cells representing vital healthy tissues. Recognition was evaluated by cytokine release measured in co-culture supernatant medium using an ELISA at 24 h. IFN-γ content was measured for most cell types, except for neurons where IL-2 content was determined. This test cell type was pre-treated with IFN-γ to induce HLA-A2 expression, precluding use of IFN-γ ELISA as readout due to high residual background values.

HLA-A2 surface expression of panel cells was confirmed by HLA-A2-specific antibody binding in flow cytometry (Supplementary Figure S1A). A positive control for effector cell recognition consisted of CD8+ T cells expressing a TCR that recognized HLA-A2 as an allo-antigen, independent of specific peptide (i.e., allo-A2 T cells). As all test panel cells were HLA-A2-positive, this effector cell control was used to demonstrate that every test cell was able to induce cytokine secretion upon effector cell recognition, irrespective of exogenous VLD.

**Figure 3:**
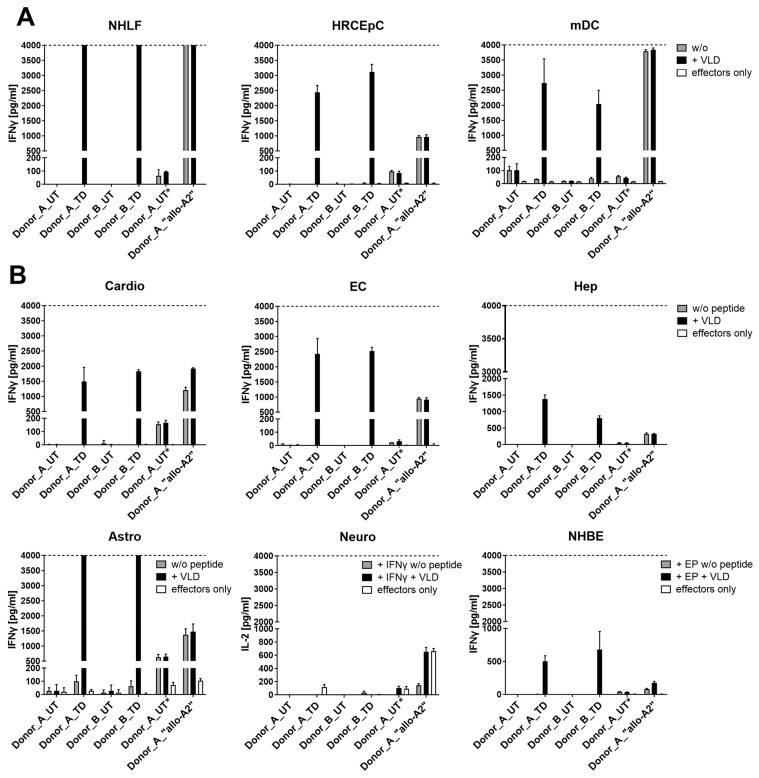
No (cross-)recognition of healthy cells by MDG1011. (**A**) IFN-γ release (pg/mL) of VLD-TCR-T cells (TD) after 24 h of co-culture with PRAME mRNA-positive normal human lung fibroblasts (NHLF), human renal cortical epithelial cells (HRCEpC), and mature dendritic cells (mDC). (**B**) IFN-γ release or IL-2 release (Neuro) (pg/mL) of VLD-TCR-T cells (TD) after 24 h of co-culture with PRAME mRNA-negative cardiomyocytes (Cardio), endothelial cells (EC), hepatocytes (Hep), astrocytes (Astro), IFN-γ pre-treated neurons (Neuro), and normal human bronchial epithelial cells (NHBE) electroporated with HLA-A*02:01 *ivt*RNA (NHBE + EP). UT cells and T cells alone served as negative controls. Healthy cells loaded externally with the PRAME VLD and allo-A2 T cells served as positive controls. Shown are mean values of triplicates with standard deviations. * Due to a slightly modified expansion protocol for “allo-A2” TCR-T cells, an additional UT control was generated for Donor_A to match this condition.

Three of the nine test cell panel members expressed PRAME mRNA (Figure S1B). These were candidate cells for on-target/off-tumor recognition. Allo-A2 T cells recognized all three cell types. In contrast, MDG1011 cells were not triggered by untreated test cells, but strong IFN-γ induction was seen when they were pulsed with exogenous VLD (Figure 3A). UT effector cells did not recognize VLD-pulsed test cells, demonstrating the need for VLD-TCR expression for recognition.

Six additional panel cells representing other vital healthy tissues did not express PRAME mRNA (Supplementary Figure S1B). Allo-A2 T cells recognized all test cells, confirming adequate HLA-A2 expression and capacity to induce cytokine secretion by relevant effector cells (Figure 3B). Once again, MDG1011 cells recognized VLD-pulsed test cells, but recognition did not occur in absence of exogenous peptide. Notably, evaluation of killing of healthy cells using the IncuCyte ZOOM live-cell imaging system confirmed lack of (cross)-recognition of healthy cells by MDG1011 (internal unpublished observations). Thus, there were no signs of off-target toxicity directed against this panel of test cells representing vital healthy tissues.

### 3.2. Characterization of MDG1011 Investigational Medicinal Products (IMPs)

Based on the key 3S attributes established in the preclinical studies of MDG1011, an ACT approach was investigated in an FIH clinical study of MDG1011 TCR-T therapy. Thirteen IMPs generated for different patients with R/R AML, MDS, or MM are shown in Table 2. The IMPs were generated from predominantly elderly patients with advanced disease, including patients with significantly elevated tumor blast cell counts in the starting apheresis products, which renders the production of pure, viable drug products very challenging.

#### 3.2.1. Cellular Composition of Starting Materials and Final MDG1011 IMPs

**Figure 4:**
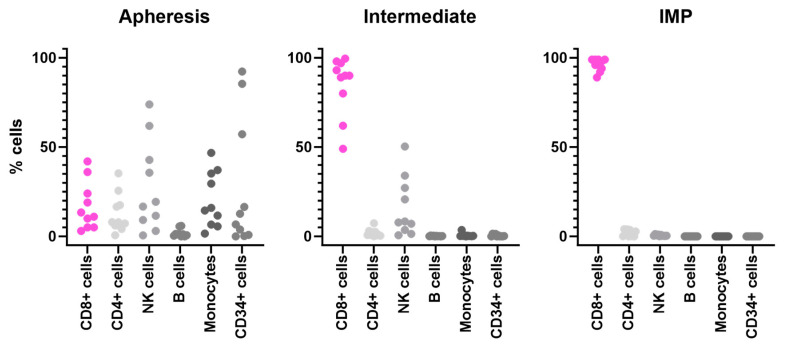
Cellular composition of MDG1011 starting apheresis products, intermediate products, and IMPs. Cells derived from ten patients were labeled with specific fluorochrome-conjugated antibodies followed by flow cytometric analysis. Frequencies of CD8+ T cells (pink, CD3+/CD8+), CD4+ T cells (CD3+/CD4+), NK cells (CD3−CD56/CD16+, B cells (CD19+), monocytes (CD14+), and CD34+ cells (tumor blasts) within viable leukocytes are shown for apheresis products (**left graph**), intermediate products (**middle graph**), and IMPs (**right graph**).

The cellular composition of apheresis products evaluated for ten patients was highly variable, but all starting materials contained the expected populations of CD4 and CD8 T cells, NK cells, B cells, and monocytes. CD34+ tumor blasts were often detected in starting materials, exceeding 50% in several apheresis products (Table 2 and Figure 4, left panel). Following CD8 enrichment, the initial cellular complexity was strongly reduced in intermediate products, yielding dominant fractions of CD8+ T cells in all cases and lower fractions of double-positive CD4+CD8+ T cells and CD8+ NK cells; B cells and monocytes were mostly eliminated (Figure 4, middle panel). The CD8 enrichment process was very effective in eliminating CD34+ cells, even when they represented more than 50% of starting cells. Intermediate cells were transduced to express VLD-TCRs and expanded for eleven days, using proprietary methods, yielding final IMPs with highly pure fractions of CD8+ T cells (Figure 4, right panel).

**Figure 5:**
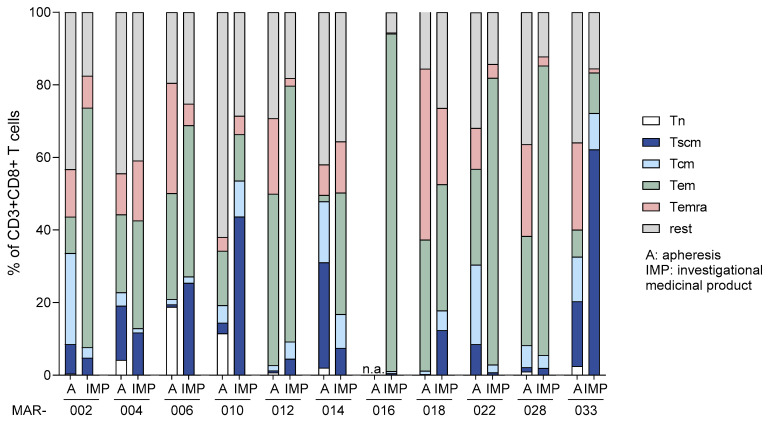
T memory cell composition of starting apheresis materials and IMPs. Cells obtained from apheresis products (A) and IMPs derived from eleven patients were stained with specific fluorochrome-conjugated antibodies and subsequently analyzed using flow cytometry. The data illustrate the frequencies of different T memory subsets among viable CD3+/CD8+ cells, with the following color coding: white for naïve (Tn, CD45RA+/CCR7+/CD27+/CD95−), blue for stem cell memory (Tscm, CD45RA+/CCR7+/CD27+/CD95+), light blue for central memory (Tcm, CD45RA−/CCR7+/CD27+), green for effector memory (Tem, CD45RA−/CCR7−/CD27−), rose for effector memory RA+ (Temra, CD45RA+/CCR7−/CD27−), and gray for remaining cells; n.a.—not analyzed.

Further phenotype analyses compared CD8+ T cells of apheresis products with the corresponding IMPs after cryopreservation at the end of manufacturing and thawing, as intended for patient infusion (Figure 5). In all cases, changes in the composition of cell subpopulations were observed. Significant variability was observed in the frequencies of naïve T cells and T stem cell memory (Tscm) cells, with proportions ranging from 0.3% to 31% of the respective initial apheresis products. This variability resulted in a highly variable final T cell composition of 1–72% of T central memory (Tcm) and Tscm cells in the final IMPs. The highest fraction of these cells was seen in the single MDS patient (033) included among the eleven patient samples analyzed in this comparison.

#### 3.2.2. VLD-TCR Expression by MDG1011 IMPs

**Figure 6:**
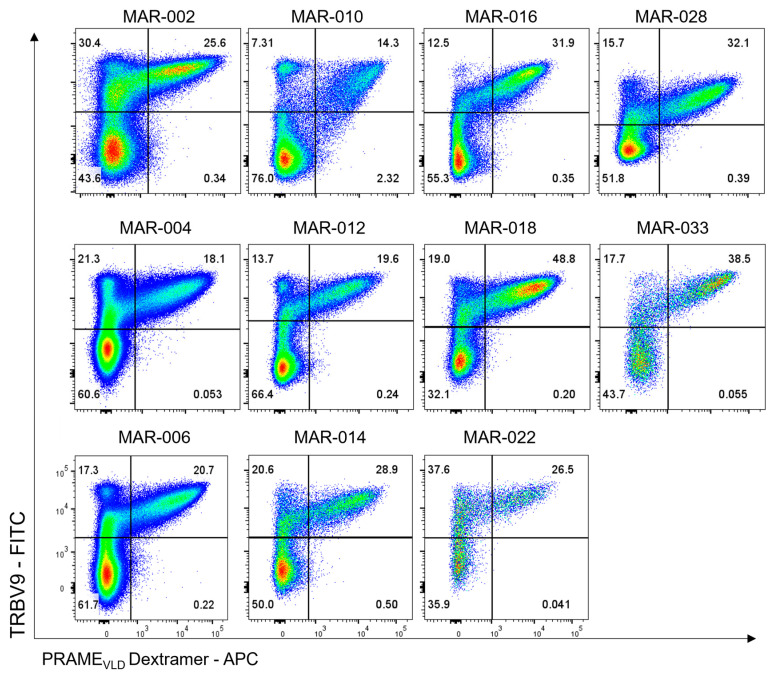
Cell surface VLD-TCR expression in MDG1011 IMPs. Cells derived from the IMPs of eleven patients were stained with specific fluorochrome-conjugated antibodies and subsequently analyzed using flow cytometry. The frequencies of MDG1011, identified using TRBV9+/PRAME VLD Dextramer+, are represented in the upper right quadrant among the gated viable CD3+/CD8+ T cells.

MDG1011 IMPs were also assessed for VLD-TCR surface expression using a dual staining approach, combining an antibody specific for the V-beta chain of the VLD-TCR (TRBV9) and a PRAME VLD-specific/HLA-A2 dextramer (Figure 6). Results are shown for eleven batches of MDG1011. In all cases, substantial fractions of double-positive cells were detected, ranging from 14 to 49% of CD3+/CD8+ T cells. Elsewhere, it was shown that higher levels of TCR expression were associated with higher VLD-TCR vector copy numbers (VCN), using sorted subfractions of single- and double-positive cells prepared from healthy donors. Thus, variations in VCN in individual cells likely contributed to the staining variations for both markers seen in the flow cytometry assessments of these MDG1011 IMPs.

#### 3.2.3. Functional Assessment of MDG1011 IMPs

Experiments were performed to assess the functional capacity of these eleven IMPs to secrete IFN-γ and mediate killing in co-cultures, including three different cell lines as APCs or target cells. T2 cells were used after pulsing with exogenous VLD, as used in the preclinical studies described above. K562 cells are leukemic cells devoid of endogenous HLA-A2 expression, but positive for intracellular PRAME protein; they were genetically modified to express HLA-A2 surface molecules. The MElanoma cell line Mel624.38 was included as a third target cell line, since adherent cells are better suited for in vitro killing assays.

All IMP batches secreted IFN-γ in co-cultures with T2 cells pulsed with VLD, K562-A2, and Mel624.38, demonstrating their functional activity against PRAME-positive target cells (Table 3). UT batches did not respond to these APCs. An assessment of MDG1011-mediated cytotoxicity showed that all IMP batches efficiently killed PRAME VLD-loaded T2 cells and the adherent tumor cell line Mel624.38. Higher differences in cytotoxic activity against K562-A2 were observed between the different IMPs, whereby two IMP batches showed suboptimal levels of target cell killing (Table 4). However, K562-A2 cells triggered similar amounts of IFN-γ in the IMPs analyzed, as seen in comparison to co-cultures with PRAME VLD-loaded T2 or Mel624.38 cells, indicating that this tumor cell line is generally recognized by MDG1011 and target cell-intrinsic characteristics other than peptide-HLA complex expression may be responsible for suboptimal killing of K562-A2 by several IMPs.

## 4. Discussion

The PRAME-specific VLD-TCR was chosen because of its salient attributes of high specificity, sensitivity, and safety. These key 3S characteristics were established in extensive preclinical studies summarized here using TCR-T cells prepared from healthy donors as the basis for initiation of a FIH clinical study. The clear specificity of the recombinant TCR for the PRAME VLD_100–108_ was demonstrated in comparison to the counter-control using PRAME SLL_425–433_ [21]. Both VLD and SLLs bind well to HLA-A2 molecules; thus, experiments with synthetic peptides were required to establish epitope specificity, as distinctions were not possible testing tumor cell lines that express endogenous PRAME protein, since both peptides can be presented simultaneously by tumor cells (unpublished observations). As expected, MDG1011 displayed identical VLD specificity for both IFN-γ secretion and cell-mediated cytotoxicity.

Studies of TCR sensitivity give indications of how well TCR-T therapies will be in recognizing malignant cells with low levels of HLA-A2 or target antigen. The sensitivity of the VLD-TCR was established by assessing its functional avidity, testing MDG1011 recognition of APCs pulsed with serial dilutions of VLD. The EC50 value fell in the range of sensitivity considered relevant for clinical efficacy [22,29]. Functional avidity is influenced by several factors: TCR affinity, levels of TCR surface expression, and TCR-T cell display of additional surface molecules, such as CD8 co-receptors and adhesion molecules that govern T cell-target cell interactions. While TCR affinity remains constant, the other parameters can vary over time as T cells become activated and undergo proliferation [30]. When MDG1011 batches were tested with different target cells, subtle variations were apparent. MDG1011 of healthy donors displayed equal sensitivities to VLD-pulsed T2 cells, where substantial target epitope was assured by provision of exogenous VLD. In contrast, cell-mediated cytotoxicity of Mel624.38 tumor cells occurred with both MDG1011 batches, but to different degrees. Here, VLD-peptide/HLA-A2 complexes at the tumor cell surface are expected to be substantially lower and more likely to be heterogenous in expression among the target cells. As TCR affinity is constant, variations in TCR expression and accessory molecules likely contributed to the differences in Mel624.38 recognition.

Knowledge of the precise PRAME epitope seen by the VLD-TCR was essential to identify other peptides with partial homology to VLD at the transcript or protein levels for safety studies of epitope cross-reactivity. This was assessed using a test panel of mismatched peptides, which were predicted by in silico tools to potentially be present on HLA-A*02:01-positive healthy tissue [28]. In earlier clinical trials, TCR-T recognition of peptides with identical sequences encoded by related genes [31] or responses to a partially homologous sequence present in the heart [32,33] led to morbidity and/or mortality, emphasizing the critical nature of comprehensive safety assessments of mismatched epitopes.

As not all potential cross-reactive epitopes can be discerned; studies of test cells representing vital healthy tissues were of high relevance to eliminate on-target/off-tumor and off-target recognition of healthy tissues by TCR-T cells. Potential signals of epitope cross-reactivity or aberrant test cell recognition must be fully de-risked before clinical development can advance to patient studies. In addition, homologies among various HLA alleles can lead to allo-HLA cross-recognition of cells that present unknown endogenous peptides by HLA molecules other than HLA-A2. Fortunately, these can be more precisely identified by examining an extensive panel of allogeneic LCL. If cross-recognition is observed for another HLA allotype, patients bearing the relevant HLA allele(s) can be excluded from participation in clinical studies and also for later eligibility in authorized treatments.

These rigorous and complex studies were essential to assure the highest levels of safety at the preclinical stage, as patients receive IMPs, which are living drugs that can be retained and are active for long periods of time in the body, and it is precisely this characteristic that is desired for efficacious treatment of rapidly progressing hematological malignancies. Fortunately, no safety signals were discerned for MDG1011, indicating an excellent safety profile for the VLD-TCR and MDG1011 based on the parameters assessed. In fact, this safety profile for MDG1011 was confirmed by findings of no dose-limiting toxicity in CD-TCR-001 clinical study patients whose IMPs are described here [23].

Numerous factors can impact the quality, quantity, and functionality of MDG1011 drug products manufactured for individual patients with R/R AML, MDS, and MM, which will not be seen using cells of healthy donors. In particular, the cellular composition of apheresis starting materials varied widely among patients, as did the resultant IMPs. It is noteworthy that these IMPs were prepared mostly from elderly patients who had undergone extensive chemotherapies. In some cases, IMPs were made for individuals with uncontrolled disease, evidenced by high CD34+ frequencies in apheresis starting materials, where negative systemic impacts on lymphocyte quality would be expected.

Levels of TCR expression and percentages of TCR-transduced cells were variable in the IMP batches. Elsewhere, TCR expression was shown to correlate directly with VCN, whereby greater surface expression was found in T cell subfractions with higher VCN. Retroviral transduction is dependent on cell proliferation; thus, variations in transgene insertions and frequences of TCR-positive cells will vary in mixed starting cell populations that are not monoclonal and not synchronized for cell cycle. Furthermore, treatment histories and systemic impacts of disease in individual patients will impose constraints on the biological properties of the T cells used for TCR-T manufacturing, contributing to variations in transduction rates and levels of TCR expression seen in the IMPs. Despite such impacts, a success rate of 92% for the manufacturing of drug products with adequate numbers of VLD-TCR-expressing TCR-T cells was achieved for patients entered in CD-TCR-001, a satisfactory finding that was not anticipated before the trial’s beginning. Furthermore, it is remarkable that all final TCR-T drug products displayed strong cytokine secretion and tumor cell killing, despite clinical indication, status, and quality of apheresis starting materials.

CAR-T studies and animal models demonstrated that the presence and prevalence of T cells with stem-like properties (i.e., Tscm and Tcm) correlate with better clinical responses [34,35,36,37]. High variations were seen in the content of these cells in the different IMPs. Interestingly, the final content was partially reflected in their content in the starting apheresis products. Recent developments indicate ACT employing a manufacturing process of shorter duration may increase these cell types, with parallel improvement in numbers of patients having clinical benefit of longer duration [38]. Given the high rate of successful production of IMP for these patients with extremely advanced disease, there is clearly room for future improvement in the MDG1011 manufacturing process to impact TCR-T cellular composition with respect to these important T cell subpopulations. Our recent research shows that shorter production times can provide TCR-T cells with much higher fractions of Tscm and Tcm.

## 5. Conclusions

The preclinical studies described here confirmed that T4.8-1-29, the VLD-TCR expressed in the T cell-based cellular product MDG1011, displayed the critical 3S attributes of specificity, sensitivity, and safety necessary for use in clinical studies. Safety and functionality of MDG1011 was evaluated using a comprehensive set of cell lines and test panels, demonstrating its favorable safety profile, as well as promising functional capabilities. This enabled regulatory approval for the FIH use of MDG1011 to treat patients suffering from three types of myeloid malignancy.

MDG1011 drug products manufactured from heavily pre-treated and mostly elderly patients showed adequate TCR transgene cell surface expression, heterogenous T memory cell composition, and high functionality upon encounter of MDG1011 with antigen-positive target cells. This substantiated the successful manufacture of CD8-enriched active TCR-T cell products from apheresis materials displaying challenging starting cell compositions, including extremely high fractions of leukemic blasts in several patients. Based on the favorable safety profiles of PRAME as a target antigen and MDG1011 in vitro and in vivo, this TCR-T therapy approach merits further investigation as a potential ACT to fill the unmet medical need for patients suffering from myeloid malignancies.

## 6. Patents

D.J.S. is a co-inventor on WO2017/216324 related to this work and is filed by Medigene Immunotherapies GmbH.

## Figures and Tables

**Table 1:** Overview of the test cell panels and assays used to assess safety of MDG1011.

Assessment	Test System	Method	Results
On-target/off-tumor toxicity	PRAME mRNA-positive healthy cells, with or without PRAME VLD	Co-cultures of MDG1011 and healthy test cells: IFN-γ secretion at 24 h Live-cell imaging assay 0–38 h	No recognition of PRAME mRNA-positive healthy cells
Off-target/off-tumor toxicity	Human LCL panel (N = 52) with common HLA allotypes: unloaded or loaded with PRAME VLD	Co-cultures of MDG1011 and LCL panel cells: IFN-γ secretion at 24 h	No recognition of unloaded LCL; four PRAME VLD-loaded LCL were recognized that expressed HLA-A*02:07, HLA-A*02:16, and HLA-A*02:17
	Mismatched peptide test panel identified using Expitope^®^ 2.0 database: 15 peptides loaded on T2 cells: 1 peptide was expressed as *ivt*RNA in tumor cell lines	Co-cultures of MDG1011 and mis-matched peptide-pulsed T2 cells or *ivt*RNA-transfected tumor cell lines: IFN-γ secretion at 24 h	One mismatched peptide cross-recognized on peptide- loaded T2 cells; no recognition of mismatched peptide-encoding *ivt*RNA in tumor cell lines
	PRAME mRNA-negative panel of primary human healthy cell types; unloaded or loaded with VLD peptide	Co-cultures of MDG1011 and healthy test cells: IFN-γ secretion at 24 h Live-cell imaging assay 0–38 h	No cross-recognition of PRAME mRNA-negative test cells of the healthy cell panel

**Table 2:** Patient indication, age, and percentage of CD34+ tumor blasts in starting materials for IMP production.

IMP	Indication	Age	% CD34^+^ Tumor Blasts in Apheresis Products ^1^
MAR-002	AML	58	85
MAR-004	MM	64	0
MAR-006	AML	67	13
MAR-010	AML	58	57
MAR-012	AML	55	1
MAR-014 ^#^	AML	77	82
MAR-016	AML	60	92
MAR-018	MM	60	0
MAR-022 ^#^	AML	65	26
MAR-024 *	AML	77	4
MAR-028	AML	65	7
MAR-030 ^#^*	AML	69	86
MAR-033	MDS	80	17

^1^ CD34+ tumor blasts were not detected in any apheresis products acquired from healthy donors. ^#^ Out of Specification batches; no cellular composition data shown in Figure 4; * No phenotype and functional data available.

**Table 3:** IFN-γ secretion of MDG1011 IMPs upon stimulation with APCs or tumor cell lines.

Secreted IFN-γ (±SD) ^1^			
IMP	T2_VLD	K562-A2	Mel624.38
MAR-002	1886 (±55)	2795 (±68)	988 (±27)
MAR-004	3443 (±237)	3420 (±173)	ND
MAR-006	1738 (±115)	3287 (±1236)	971 (±33)
MAR-010	2200 (±61)	2461 (±357)	205 (±12)
MAR-012	1771 (±83)	2093 (±557)	238 (±05)
MAR-014	772 (±19)	678 (±92)	141 (±26)
MAR-016	1526 (±44)	931 (±48)	661 (±83)
MAR-018	1928 (±44)	1434 (±80)	972 (±58)
MAR-022	455 (±11)	611 (±49)	191 (±14)
MAR-028	1673 (±200)	2016 (±69)	589 (±61)
MAR-033	293 (±19)	448 (±23)	51 (±8)

^1^ amount of secreted IFN-γ determined using ELISA after 24 h co-culture with indicated target cells, given in pg/mL (±SD); ND—not determined.

**Table 4:** MDG1011 IMP-mediated killing of APCs or tumor cell lines.

% Killing of Target Cells (±SD) ^1^			
IMP	T2_VLD	K562-A2	Mel624.38
MAR-002	ND	ND	ND
MAR-004 ^2^	63 (±2.6)	66 (±0.3)	ND
MAR-006	75 (±7.1)	12 (ND)	40 (ND)
MAR-010	66 (±1.0)	9 (±0.9)	76 (±6.1)
MAR-012	45 (±4.8)	31 (±1.4)	99 (±0.2)
MAR-014	96 (±3.3)	34 (±2.9)	32 (±20.9)
MAR-016	64 (±6.2)	9 (±3.0)	99 (±0.4)
MAR-018	85 (±2.0)	24 (±1.1)	99 (±0.5)
MAR-022	ND	ND	ND
MAR-028	99 (±0.9)	24 (±2.4)	100 (±0.1)
MAR-033	95 (±1.0)	49 (±2.0)	46 (±9.8)

^1^ normalized killing of indicated target cells after 72 h (^2^ MAR-004: 16 h) in %; ND—not determined.

## Data Availability

Data are available upon reasonable request. Data availability will be possible from D.J.S.

## Supplementary Materials

The following supporting information can be downloaded at: https://www.mdpi.com/article/10.3390/cancers17020242/s1, Figure S1: Cell surface HLA-A2 and PRAME mRNA expression in healthy cells.
