# Supplementary material for: Characterization of a 3S PRAME VLD-Specific T Cell Receptor and Its Use in Investigational Medicinal Products for TCR-T Therapy of Patients with Myeloid Malignancies
Source: Cancers (Basel). 2025 Jan 13;17(2):242. doi: 10.3390/cancers17020242 (PMC11763942; doi:10.3390/cancers17020242)
Supplement: Supplementary file 1 [file cancers-17-00242-s001.zip › cancers-3339119-supplementary.pdf]

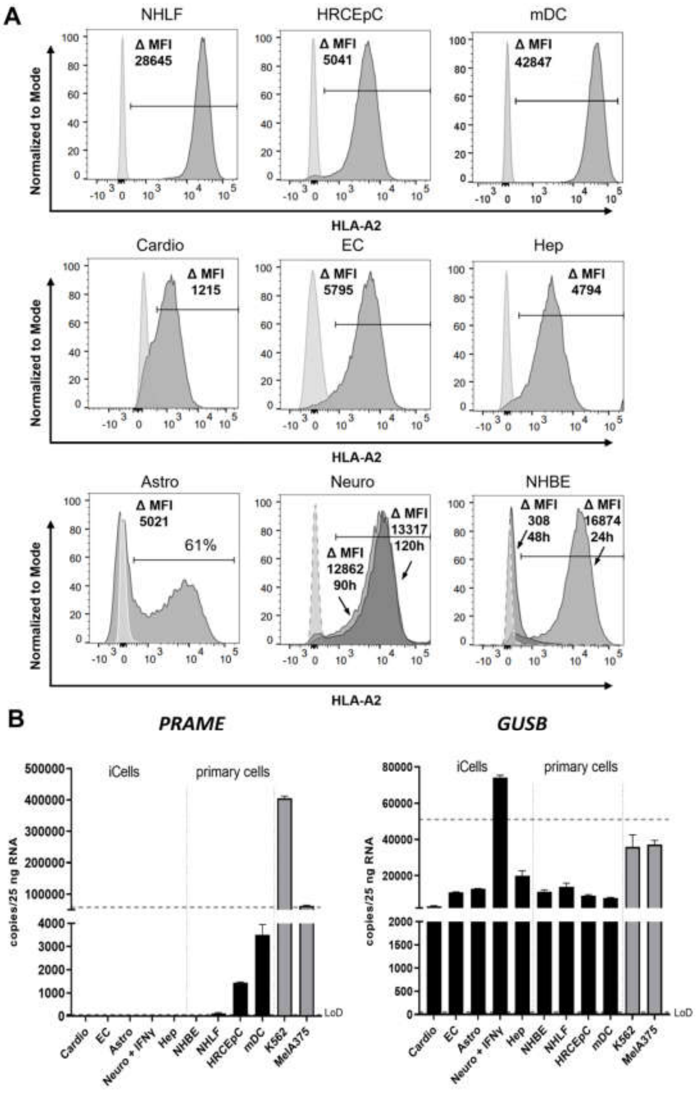

Figure S1: Cell surface HLA-A2 expression and *PRAME* mRNA expression in healthy cells. (A) Cell surface HLA-A2 expression was determined by flow cytometric analysis using a monoclonal anti-HLA-A2 antibody (clone BB7.2, BD Biosciences). Depicted are histograms and Δ MFI values. HLA-A2 expression in *PRAME*-positive normal human lung fibroblasts (NHLF), human renal cortical epithelial cells (HRCEpC) mature dendritic cells (mDC) and in *PRAME*-negative iPSC derived iCell cardiomyocytes (cardio), iCell endothelial cells (EC), iCell Hepatocytes (Hep) and iCell Astrocytes (Astro). Histograms filled with light and dark grey show un-stained and stained cells, respectively. For iCell Neurons (Neuro), HLA-A2 expression is shown after IFN-γ treatment for 90 h and further culture for 24 h without IFN-γ (total time: 120 h) (indicated by the arrows). Histograms filled with light and dark grey show specific staining of un-treated and IFN-γ-treated Neuro cells, respectively. HLA-A2 expression of bronchial epithelial cells (NHBE) is depicted at 24 h and 48 h after electroporation with 50 µg of HLA-A*02:01 *ivt*RNA (indicated by the arrows). Histograms filled with light and dark grey show staining of un-transfected and transfected NHBE, respectively. (B) *PRAME* and *GusB* mRNA expression (copies per 25 ng RNA) was determined by qPCR. Depicted are mean values of duplicates with standard deviations. Black and grey bars represent healthy cells and tumor cell lines, respectively. The dashed gray lines show the limit of detection (LoD) at 58 copies per 25 ng RNA for PRAME and 51 copies per 25 ng RNA for GusB, respectively. The LoD is defined as the copy number of the lowest concentration of the standard curve.
